# Biochemical and Microstructural Properties of Lizardfish (*Saurida tumbil*) Scale Collagen Extracted with Various Organic Acids

**DOI:** 10.3390/gels8050266

**Published:** 2022-04-24

**Authors:** Abdul Aziz Jaziri, Rossita Shapawi, Ruzaidi Azli Mohd Mokhtar, Wan Norhana Md. Noordin, Nurul Huda

**Affiliations:** 1 Faculty of Food Science and Nutrition, Universiti Malaysia Sabah, Kota Kinabalu 88400, Sabah, Malaysia; azizjaziri@ub.ac.id; 2 Faculty of Fisheries and Marine Science, Universitas Brawijaya, Malang 65145, Indonesia; 3 Borneo Marine Research Institute, Universiti Malaysia Sabah, Kota Kinabalu 88400, Sabah, Malaysia; rossita@ums.edu.my; 4 Biotechnology Research Institute, Universiti Malaysia Sabah, Kota Kinabalu 88400, Sabah, Malaysia; ruzaidi@ums.edu.my; 5 Fisheries Research Institute, Batu Maung 11960, Penang, Malaysia; wannorhana@yahoo.com

**Keywords:** lizardfish scale, organic acid-aided extraction, structural characteristic, biochemical property

## Abstract

The purpose of this research was to extract collagen from the scales of lizardfish (*Saurida tumbil*) using various acids. Acetic acid-extracted collagen (AScC) produced a higher yield (1.8 mg/g) than lactic acid-extracted collagen (LScC) and citric acid-extracted collagen (CScC) although not significantly different (*p* > 0.05). All extracted collagens were categorized as type I collagens with the presence of alpha chains (α_1_ and α_2_) based on the SDS-PAGE profiles. The triple-helical structure of the collagen was maintained in the AScC, LScC, and CScC as confirmed by the FTIR spectra. The UV-vis and X-ray diffraction spectra observed in all collagens were in agreement with previous work on fish scale and calfskin (commercial) collagens. The thermal stability of AScC (*T_max_* = 31.61 °C) was greater than LScC (*T_max_* = 30.86 °C) and CScC (*T_max_* = 30.88 °C). The microstructure of acid-extracted collagens was characterized as complex, fibrous, and multilayered, with irregular sheet-like structures. All samples were highly soluble in acidic pH (1.0–4.0) and in low concentrations of NaCl (0–20 g/L). In conclusion, the lizardfish scale collagen, particularly AScC, may be used as an alternative to terrestrial animal collagen.

## 1. Introduction

Collagen is a fibrous protein that contains a unique right-handed triple-helix structure composed of three parallel, left-handed polypeptide chains. Each chain is governed by a Glycine-X-Y repeating sequence where X and Y are often proline and hydroxyproline [1,2]. This fibrous protein plays an important role in connective tissues, such as tendons, ligaments, skin, and bones. It is also considered the most abundant protein, making up almost 30 percent of the total protein composition in vertebrates [3]. Approximately 29 types of collagens have been reported based on their protein structure, amino acid sequence, and molecular properties [2]. Among collagens, type I is extensively applied in many industrial sectors including foods, cosmetics, biomedicine, pharmaceuticals, and nutraceuticals due to its superior traits, such as biodegradability, biocompatibility, and weak antigenicity [4]. Traditionally, commercial collagens were produced from terrestrial animals (bovine and porcine). However, a mass outbreak of diseases such as bovine spongiform encephalopathy, transmissible spongiform encephalopathy, and foot-and-mouth disease in cattle and pigs, have discouraged manufacturers and consumers [5]. In addition, Muslims and Jews are prohibited to consume porcine-derived products, and bovine-based products are not acceptable for consumption by Hindus and Sikhs [6]. To solve these issues, alternative sources of collagen need to be studied.

Collagen from fish has gained attention from many researchers because of its comparable properties to the collagen from terrestrial animals [7]. In addition, it is acceptable to most religious beliefs. Moreover, fish collagen is usually produced from the by-products (such as skin, bone, and scale) from fish-processing industries which could bring additional profits. Many studies have demonstrated the extraction of collagen from the skin, bones, and scales of bigeye tuna (*Thunnus obesus*) and grass carp (*Ctenopharyngodon idellus*) [8,9], the skin and bones of Spanish mackerel (*Scomberomorous niphonius*) and lizardfish (*Saurida tumbil*) [10,11,12], the skin of sturgeon fish (*Huso huso*), barramundi (*Lates calcarifer*), and tilapia (*Oreochromis niloticus*) [13,14], and the scales of grey mullet (*Mugil cephalus*), giant grouper (*Epinephelus lanceolatus*), carp (*Cyprinus carpio*), and miiuy croaker (*Miichthys miiuy*) [15,16,17,18]. In addition, the physicochemical characteristics of the collagens, including solubility, thermostability, molecular weight, as well as structural analysis, have also been evaluated. Collagen extracted by adding acids is known as the acid-aided technique [19]. Organic acetic, lactic, and citric acids are often used in collagen extraction because they are more effective than inorganic acids [20], particularly in dissolving the non-crosslinked components and breaking the inter-strand crosslinks in collagen, which leads to the very high solubility of collagen during the extraction process [21].

Lizardfish (*Saurida tumbil*) is an economically important tropical marine fish species in Malaysia. It is commercially used as a raw material in surimi processing due to its strong gel-forming ability, high-yield production, and white flesh [22]. Adult lizardfish have body length sizes ranging from 19 cm to 35 cm. The dorsal part of the body is brown in color. The ventral part is silver in color with black, faint crossbands [23]. The production of lizardfish in Malaysia from 2015 to 2019 amounted to approximately 48,153 metric tons [24]. In general, about 60–75% of the total weight of raw fish is generated as by-products from surimi processing. These by-products are usually turned into low-value goods, such as fertilizer, fish meal, animal feed, and biodiesel [25]. Sometimes the by-products are discarded as waste and pollute the environment. This could lead to a significant loss of potential revenue and additional costs for the disposal of the by-products [23]. A high-value product needs to be generated from these valuable by-products that could result in financial gain and produce zero waste.

The nutritional value of lizardfish by-products has been documented and shows promising worth. Interestingly, a higher protein content (29.62%) was reported in the scales compared to the bones, fins, and spare flesh [26], suggesting that scales are a prospective source of collagen. Other researchers have extracted type 1 collagen from the scales of other fish species [27]. Research on the extraction of collagen from lizardfish (*S. tumbil*) scales is limited in terms of the extraction media and the quality characteristics of the end products. Therefore, this study aimed to extract collagen from the scales of lizardfish with three types of organic acids and to determine its microstructural and physicochemical characteristics.

## 2. Results and Discussion

### 2.1. Yield and Hydroxyproline (Hyp)

Table 1 shows the yield, Hyp, and total collagen from lizardfish scales extracted with different acids. The yields of all acid-extracted collagens (AScC, LScC, and CScC) based on the wet weight of the lizardfish scales were low (<0.2 g/100 g). Our readings are 2–4 times lower than the yields of collagens from seabass (*L. calcarifer*) (0.38 g/100 g) [27], spotted golden goatfish (0.46 g/100 g) (*Parupeneus heptacanthus*) [28], miiuy croaker (0.64 g/100 g) (*M. miiuy*) [18], and tilapia (0.77 g/100 g) (*O. niloticus*) [29] scales. The reason could be that the scales used in the previous works were based on the dry weight rather than the wet weight, as in this study. In addition, the yield of lizardfish scale collagens was also lower than those of lizardfish bone collagens (1.73–2.59 g/100 g) [11] and skin collagens (11.39–11.73 g/100 g) [12] extracted with a similar procedure. These results were in line with the study of the collagens derived from bigeye tuna (*T. obesus*) scales, bones, and skin [8]. Moreover, the yield of collagen from the scales was also much lower than that from the skin of sturgeon fish (9.98 g/100 g) [13], Spanish mackerel (13.68 g/100 g) [10], and bigeye snapper (10.94 g/100 g) [30]. This could be due to the fact that fish scales are biocomposites of highly-ordered type I collagen fibers with several cross-linked regions and hydroxyapatite (Ca_5_(PO_4_)_3_OH) [31] compared to the fish skin. In general, the AScC sample showed a higher yield than LScC and CScC, although not significantly different (*p* > 0.05). Perhaps in future work, pepsin could be incorporated to increase the collagen extraction from scales as pepsin could cleave specifically at the telopeptide region of the collagen [32].

Hydroxyproline (Hyp) is the main component of the imino acid that stabilizes the triple-helical structure of collagen. The Hyp content represents the amount of collagen since it is present exclusively in collagen [30]. The Hyp contents of all extracted collagens varied from 78.39 ± 0.11 mg/g to 83.29 ± 0.42 mg/g. AScC had a significantly higher (*p* < 0.05) Hyp compared to LScC and CScC. The Hyp compositions in the present study were lower than in the commercial collagens [18], and the collagens from the scales of other fish species such as bigeye tuna (*T. obesus*) [8], seabass (*L. calcarifer*) [29], miiuy croaker (*M. miiuy*) [18], and tilapia (*O. niloticus*) [29] (Table 1). Also, the Hyp content of lizardfish scale collagen was lower compared to that of the collagens from the bones and skin of lizardfish [11,12]. Total collagen was derived by multiplying Hyp with a conversion factor of 7.7 as described by Kittiphattanabawon et al. [30]. These results confirmed that the higher content of collagen obtained in this study was consistent with the higher composition of Hyp found in the extracted samples. The variations in the Hyp contents and yields observed were affected by several factors, such as species, size, age, structure, and composition of the fish tissue, as well as the extraction methods [33].

### 2.2. Color of Acid-Extracted Collagens

Color is an important property of collagen. Lighter-colored collagen is preferred as it does not impart an obvious color to the finished products [34]. The color attributes of the collagens extracted from the scales of lizardfish (*S. tumbil*) with acetic, lactic, and citric acids are tabulated in Table 2. Statistical analyses indicated that there was a highly significant effect (*p* < 0.05) of acids used on color attributes (i.e., L*, a*, b*, and WI). The L* value in AScC was higher than in LScC, CSkC, and CScC, whereas higher a* and b* values, which represent redness/greenness and yellowness/blueness, respectively, were observed in LScC. AScC had the greatest whiteness index (WI) compared to others. The results suggest that the use of acetic acid may increase the L* and WI of fish scale collagen. Similarly, our previous findings on the acetic acid-aided extraction of lizardfish bone collagen had the highest L* value (88.54 ± 0.42) and WI value (85.29 ± 0.10) [11]. In comparison to other fish sources, acid-extracted lizardfish scale collagens in the present study were lighter and whiter than the acid-extracted collagen from barramundi (*L. calcarifer*) skin [35]. The collagen obtained from the H_2_O_2_-treated snakehead (*Channa argus*) skins [36] is, however, lighter and whiter than our findings. The use of H_2_O_2_ might have caused this obvious difference.

### 2.3. SDS-PAGE Profile

**Figure 1:**
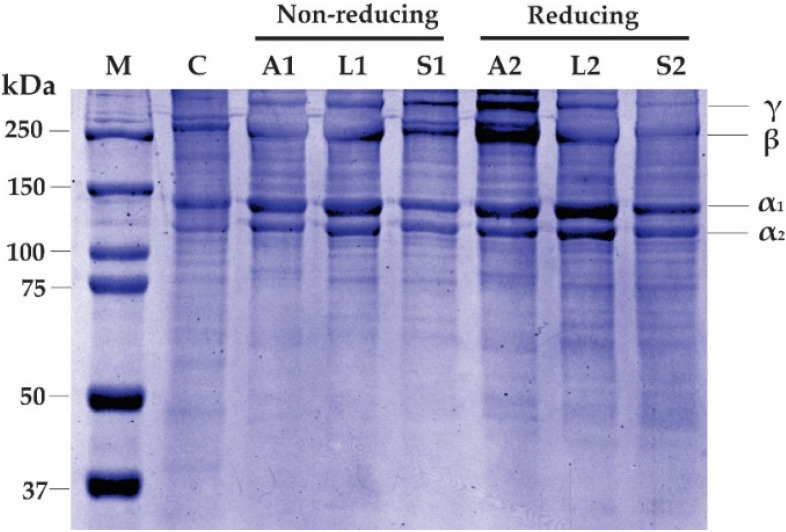
SDS-PAGE electrophoretogram of collagens from the scales of lizardfish shows the occurrence of band patterns of α, β, and γ isomers. M: collagen from calfskin; M: protein marker; A1 & A2: acetic acid-extracted collagen (AScC); L1 & L2: lactic acid-extracted collagen (LScC); S1 & S2: citric acid-extracted collagen (CScC).

Figure 1 shows the SDS-PAGE profile of AScC, LScC, and CScC from lizardfish treated under reducing and non-reducing conditions. Generally, the electrophoretic patterns of lyophilized AScC, LScC, CScC, and even CSkC were similar and composed of two alpha chains (α_1_ and α_2_), as well as β- and γ-chains. For α-chains, their molecular weights (MW) were estimated at 141.5 kDa (α_1_) and 118.9 kDa (α_2_), respectively. Due to the presence of two α chains, all the extracted samples were classified as a type I collagen [27,37]. These findings were in agreement with previous reports on the lizardfish bone and skin collagens [11,12], as well as other fish scale collagens from tilapia (*O. niloticus*) [29], spotted golden goatfish (*P. heptacanthus*) [28], seabass (*L. calcarifer*) [27], sheepshead seabream (*Archosargus probatocephalus*) [38], horse mackerel (*Trachurus japonicus*) [15], and sardinella (*Sardinella fimbriata*) [39]. The estimated electrophoretic positions of the β- and γ-chains were 260.1 kDa and 416.6 kDa, respectively, and both chains represented dimer and trimer forms. However, the band intensity of acid-extracted collagens was higher than that of calfskin collagen. It could be suggested that the lizardfish scale collagen had a higher proportion of intra- and inter-crosslinks [32]. Under reducing (with β-ME) and non-reducing (without β-ME) treatments, no different electrophoretic patterns were found in AScC, LScC, and CScC, indicating the absence of disulphide bonds, as reported in the aforementioned studies.

### 2.4. UV-Vis Spectra

**Figure 2:**
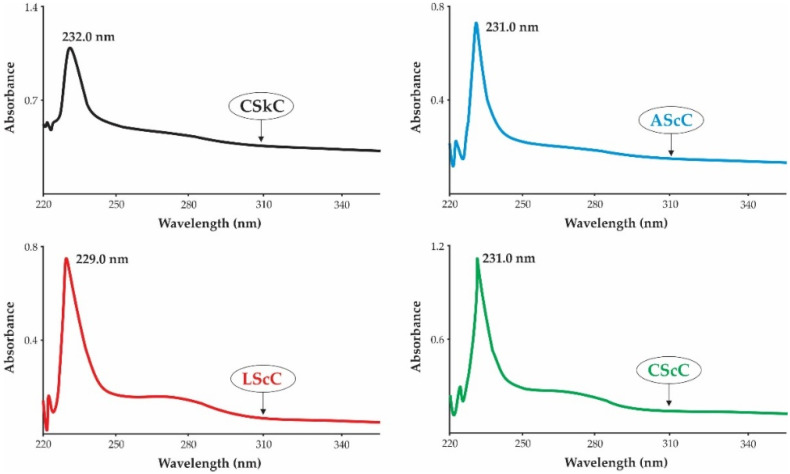
UV-vis spectra of collagens from the scales of lizardfish. AScC: acetic acid-extracted collagen; LScC: lactic acid-extracted collagen; CScC: citric acid-extracted collagen; CSkC: commercial calfskin collagen.

Collagen generally has a maximum absorption between 210 nm and 240 nm under UV-Vis Spectra [40]. The UV-vis absorption patterns of AScC, LScC, and CScC from lizardfish scale are illustrated in Figure 2. The maximum absorption peaks of acid-extracted collagens were between 229.0 and 231.0 nm, which was slightly lower than calfskin collagen (type I) (232.0 nm). These results indicated that the maximum peaks observed in both samples were closely related to the functional groups of carbonyl (C=O), carboxyl (-COOH), and amide (CONH_2_) belonging to the polypeptide chains of collagen [41]. In contrast, the low absorption peaks detected in the range of 250 nm to 300 nm, represent aromatic amino acids, such as phenylalanine, tryptophan, and tyrosine. The UV-absorption peaks of lizardfish scale collagen in the present study were in accordance with the collagen from miiuy croaker (*M. miiuy*) [18], red drum (*Sciaenops ocellatus*) [42], northern pike (*Esox lucius*) [43], and pufferfish (*Lagocephalus inermis*) [44]. Previously, our data on lizardfish (*S. tumbil*) bone and skin collagen showed a significant absorption peak within the range of 230.0–231.9 nm [11,12].

### 2.5. FTIR Spectra

**Figure 3:**
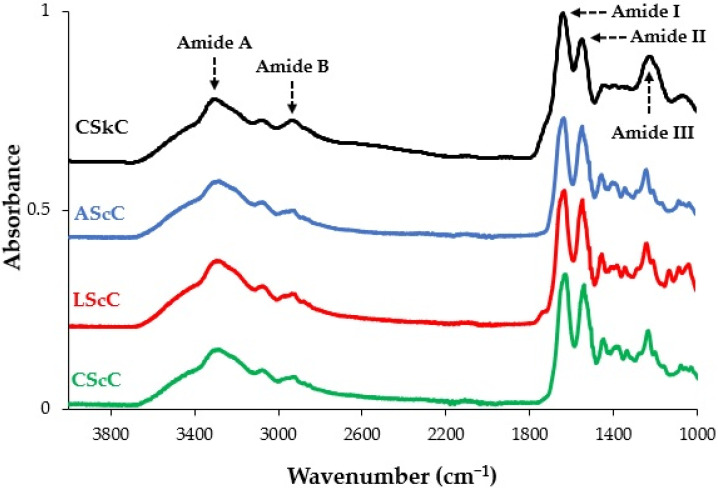
FTIR spectra of acid-extracted collagens from the scales of lizardfish. AScC: acetic acid-extracted collagen; LScC: lactic acid-extracted collagen; CScC: citric acid-extracted collagen; CSkC: commercial calfskin collagen.

The FTIR spectra of the lyophilized collagens extracted with acetic acid, lactic acid, and citric acid were evaluated, and their spectra were comparable to the commercial collagen from calfskin, as displayed in Figure 3. The characteristic peaks of amide A, amide B, amide I, amide II, and amide III were found in acid-extracted collagens (AScC, LScC, and CScC) and calfskin collagen (CSkC). These amide regions were also reported in collagens from the bone and skin of lizardfish (*S. tumbil*) under similar acid-aiding extractions [11,12], and other collagens extracted from tilapia (*O. niloticus*) [45], seabass (*L. calcarifer*) [27], grass carp (*C. idellus*) [9], as well as spotted golden goatfish (*P. heptacanthus*) [28] scales. The peak assignment of amine A is associated with an N-H stretching vibration, and the peak locations of AScC, LScC, CScC, and CSkC were detected at wavenumbers of 3276.41, 3296.91, 3285.73, and 3309.01 cm^−1^, respectively (Table 3). These wavenumbers were under the ranges (3400–3440 cm^−1^) of a free N-H stretching vibration as recommended by Doyle et al. [46], which stated that when the NH group is involved with hydrogen bonds in a peptide chain, the position is shifted to a lower frequency. For amide B, the peak locations of all acid-extracted collagens from lizardfish scales and calfskin collagen were located at wavenumbers between 3071.40 cm^−1^ and 3088.18 cm^−1^. Amide B is characterized as an asymmetrical stretch of CH_2_ [47]. Furthermore, the amide (I, II, and III) regions are involved in the formation of a triple-helical structure, representing C=O stretching, N-H bending, and C-H stretching, respectively [48]. Data showed that the amide I and II of extracted collagens were similar at wavenumbers 1628.89 and 1541.29 cm^−1^, respectively, whilst the amide III bands of lizardfish skin collagens were slightly different, particularly observed in the CScC sample (Table 3). In addition, the amide I-III bands of standard collagen were detected at higher wavenumbers of 1635.87, 1542.71, and 1237.15 cm^−1^. The higher the wavenumber of these amide regions, the weaker and/or fewer the H bonds in CSkC. At the same time, increased hydrogen bonding in the triple-helical structure of extracted collagen could result in a lower degree of molecular order in the collagen. Another approach is to verify the triple-helical structure of collagen by measuring the difference in the wavenumber (cm^−1^) between amide I and II bands using the formula Δ*v* (*v_I_* − *v_II_*), where values < 100 indicate that the triple-helical structure has been maintained [49]. The Δ*v* values of AScC, LScC, and CScC in this study were below 100, ie., 87.59, 87.59, and 87.60, respectively, suggesting that the triple-helical structures of collagens extracted from the scales of lizardfish were maintained. Furthermore, the triple helical structure could be verified by using the absorption ratio (>1.0) of the amide III to the 1450 cm^−1^ band (AIII/A1450) [50]. The results confirmed that the triple-helix structure was maintained, with an absorption ratio of 1.17 which is higher than 1.0. All the absorption peaks observed in the lizardfish scale collagens were consistent with the collagen from tilapia (*O. niloticus*) scales [45], seabass (*L. calcarifer*) scales [27], bigeye tuna (*T. obesus*) skin [8], and sharpnose stingray (*Dasyatis zugei*) skin [51].

### 2.6. X-ray Diffraction (XRD) Analysis

**Figure 4:**
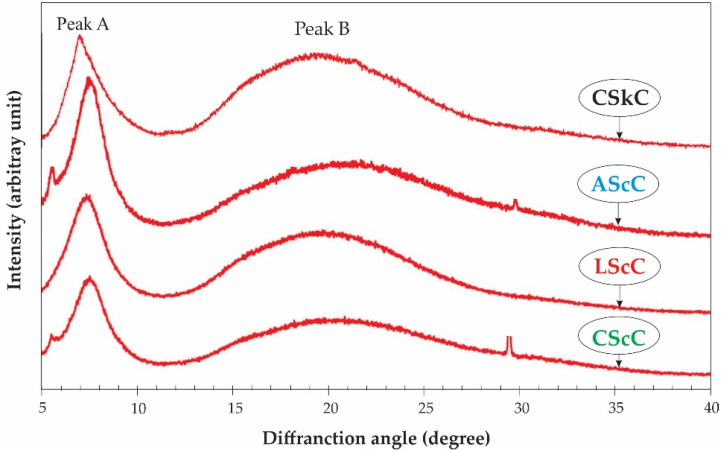
X-ray diffraction diagram of acid-extracted collagens from the scales of lizardfish. AScC: acetic acid-extracted collagen; LScC: lactic acid-extracted collagen; CScC: citric acid-extracted collagen; CSkC: commercial calfskin collagen.

XRD diagrams of the lizardfish scale collagen extracted with acetic, lactic, and citric acids were determined together with the calfskin collagen as standard. Both the acid-extracted collagens and the calfskin collagen had two prominent diffraction peaks, located at diffraction angles (2*θ*) of 7.34–7.50° and 19.65–20.69°, and 7.03° and 19.39°, respectively (Figure 4). Generally, the first peak, labeled A, was sharp, whereas the second peak (B) was broad. The two diffraction peaks characterized a collagen triple-helical as exhibited in the standard. Our previous work on the lizardfish (*S. tumbil*) skin collagens under the same extraction conditions also had similar diffraction peaks [12], and these studies have been confirmed in other sources, including carp (*C. carpio*) scale collagen [52] and tilapia (*O. niloticus*) skin collagen [53,54]. To determine the minimum value of the repeated spacings (d (Å)), the Bragg equation d(Å) = λ/2sin *θ* (where λ is the X-ray wavelength (1.54 Å), and *θ* is the Bragg diffraction angle) was used [52]. The d values of the first peak, indicating the distance between the molecular chains of the triple-helical structures in the collagens, were 11.89–11.93 Å for all extracted collagens, which were slightly lower compared to that of the standard collagen (12.00 Å). Meanwhile, the values of d at the second peak were between 4.29 Å and 4.52 Å, lower than that observed in the standard (4.58 Å), and this peak reflects the distance between the skeletons. These findings are in accordance with the diameter of a collagen molecule with a triple-helix structure and a single left-handed helix chain. Overall, all extracted collagens were in their native conformations and undenatured.

### 2.7. Thermal Stability

**Figure 5:**
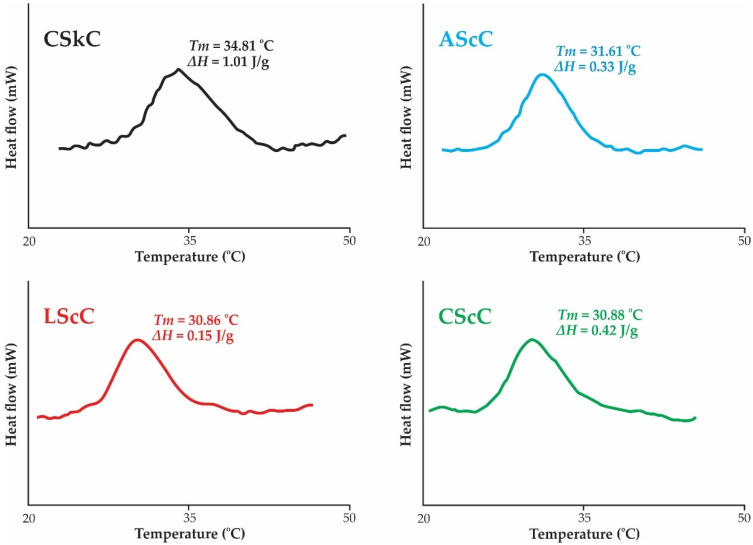
DSC thermogram of collagens from the scales of lizardfish. AScC: acetic acid-extracted collagen; LScC: lactic acid-extracted collagen; CScC: citric acid-extracted collagen; CSkC: commercial calfskin collagen.

The thermal stability of different acid-extracted collagens from the scales of lizardfish was successfully determined, and the maximum transition temperature (*T_max_*) and transition enthalpy (Δ*H*) are presented in Figure 5. A higher *T_max_* value (31.61 °C) was recorded in the AScC sample, followed by CSsC (30.88 °C) and LScC (30.86 °C); however, their values were much lower compared to the calfskin collagen (34.81 °C). The reason might be due to the higher Hyp content in CSkC (Table 1). The thermal stability of the collagen triple-helical was boosted by the pyrrolidine rings of the imino acid which were partially formed by the hydrogen (H) bonding through the hydroxyl group of hydroxyproline [37]. Moreover, Hyp stabilizes the triple-helix structure of collagen via hydrogen bonding in coil-coiled α chains [55]. Comparatively, the thermal stability of other fish-scale-derived collagens varied greatly, for example, silver carp (*Hypophthalmichthys moltrix*) (29 °C) [56], horse mackerel (*T. japonicus*) (28.1 °C), flying fish (*Cypselurus melanurus*) (29.2 °C) [15], bigeye tuna (*T. obesus*) (32.07 °C), and miiuy croaker (*M. miiuy*) (32.2 °C) [18]. In particular, collagens extracted from tropical fish species had relatively high thermostability, such as carp (*C. carpio*) scale collagen (32.8 °C) [52] and seabass (*L. calcarifer*) scale collagen (38.17 °C) [27], with Hyp contents of 87 mg/g and 85 mg/g, respectively. For the *ΔH* value, narrow areas under the peaks were observed in the acid-extracted collagen from lizardfish scales in comparison with the calfskin collagen, indicating the low energy required to uncouple the α-chains of acid-extracted collagen and convert them into random coils. Nevertheless, the difference in the thermal stability and transition enthalpy of fish collagen depends on the imino acid composition, extraction step, and other environmental factors (habitat and temperature) [30].

### 2.8. Microstructure Analysis

**Figure 6:**
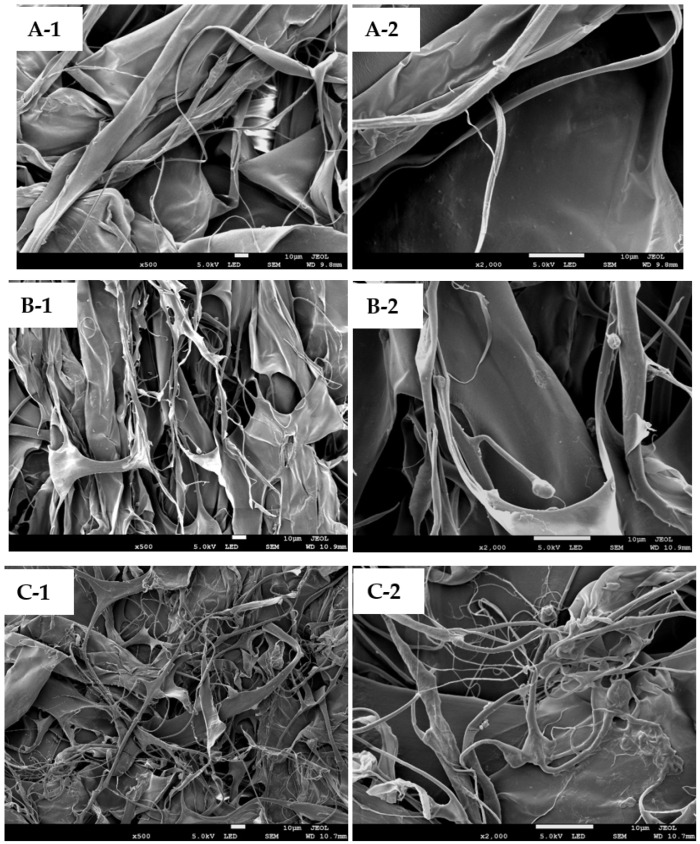
FESEM images of lizardfish scale collagens extracted with different acids. (**A**) AScC: Acetic acid-extracted collagen, (**B**) LScC: Lactic acid-extracted collagen, and (**C**) CScC: citric acid-extracted collagen. **1**: (×500); **2**: (×2000).

Under field emission scanning electron microscopy (FESEM) at a magnification of 500× and 2000×, the morphological structures of lyophilized collagens derived from the scales of lizardfish were evaluated. All samples (AScC, LScC, and CScC) showed multilayered and irregular, dense sheet-like films linked by random-coiled filaments. Fibrillar and tubular structures were also observed in the acid-extracted collagens. The loose, porous, and wrinkled structures were clearly visible (Figure 6(A-1,B-1,C-1)). This might be due to dehydration during freeze-drying as observed previously [57]. The morphology of the lizardfish scale collagens was similar to the collagens from miiuy croaker (*M. miiuy*) [18], black ruff (*Centrolophus* *niger*) [58], marine eel-fish (*Evenchelys* *macrura*) [59], and silver catfish (*Pangsius* sp.) [39]. Understanding the microstructure of collagen products is an important step for the application of collagen from pharmaceutical and medical perspectives. Previous studies have demonstrated that collagens with interconnectivity and fibrillary and sheet-like film structures could be used as a potential material in the development of coating application, new tissue formation, cell-seeding, growth, wound healing, and mass transport and migration [4,60].

### 2.9. Solubility

**Figure 7:**
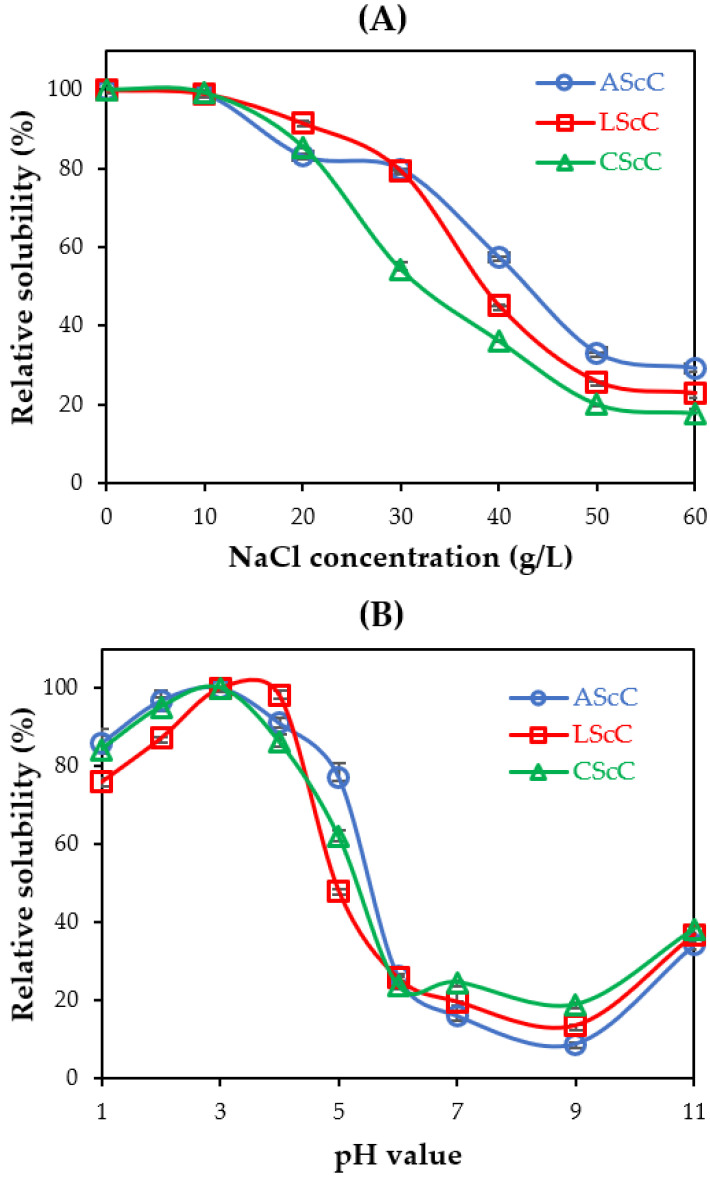
Relative solubility (%) of collagens from the scales of lizardfish (**A**) at different NaCl treatments and (**B**) at different pH conditions. AScC: acetic acid-extracted collagen; LScC: lactic acid-extracted collagen; CScC: citric acid-extracted collagen.

The solubility results for AScC, LScC, and CScC are shown in Figure 7. An analysis of the effect of different NaCl concentrations (0–60 g/L) on the extracted collagens indicated that high solubility values (>80%) were clearly detected at low NaCl treatments (0–20 g/L). The solubility of all collagens gradually decreased when 30 g/L to 60 g/L of NaCl was added. The findings suggested that at a high NaCl concentration, low solubility was obtained which could be affected by the precipitation process of the collagen when added to a high amount of NaCl. The increase in salt concentration, hydrophobic-hydrophobic interactions within the polypeptide chains, and the competition for water subsequently generated protein precipitation [45]. The relative solubilities of collagens extracted from lizardfish scales were similar to the collagens from lizardfish bone and skin [11,12], spotted golden goatfish (*P. heptacanthus*) scales [28], carp (*C. carpio*) scales [52], and miiuy croaker (*M. miiuy*) scales [18]. For pH treatments, all acid-extracted collagens showed high solubility (>70%) at acidic conditions between pH 1.0 to 4.0, particularly at pH 3.0 which denotes the highest solubilization. The solubility sharply decreased at pH close to neutral (pH 6.0) and neutral pH (7.0). The lowest solubilization was recorded at pH 9.0. It could be suggested that there is an increase in the hydrophobic-hydrophobic interactions among the collagen molecules, and the total net charge subsequently becomes zero, especially at the isoelectric point (pI) that is generally detected at slightly acidic and neutral conditions [19]. Fish scale collagens from seabass (*L. calcarifer*) [27], spotted golden goatfish (*P. heptacanthus*) [28], and bigeye tuna (*T. obesus*) [8], as well as our previous reports on lizardfish bone and skin collagens [11,12], have similar patterns of relative solubility. These results provide important information for industrial applications.

## 3. Conclusions

Collagen (type I) from the scales of lizardfish was obtained using acetic, lactic, and citric acid-assisted extraction. A higher yield of collagen was found in the acetic-extracted collagen (AScC) although not significantly different from the other acid types used (*p* > 0.05). Moreover, AScC exhibited a greater *T_max_* value compared to LScC and CScC, suggesting that it was more stable in high thermal conditions. This might be due to the high content of hydroxyproline. The triple-helical structure of collagens extracted from lizardfish scales was still preserved upon confirmation by the XRD and FTIR analyses. Overall, AScC is more selectable and could be used as an alternative material for land-based animals.

## 4. Materials and Methods

### 4.1. Chemicals

Chemicals used in this study were of analytical grade. The molecular weight markers used prestained natural protein standards (dual color standards), with a size of 10–250 kDa, and acrylamide powder was obtained from Bio-Rad Laboratories (Hercules, CA, USA). *N,N,N′,N′*-tetramethyl ethylene diamine (TEMED), sodium dodecyl sulphate (SDS), Coomassie Blue R-250, Folin-Ciocalteu’s phenol reagent, Lowry reagent, and acetic acid (CH_3_COOH) were purchased from Merck (Darmstadt, Germany). Tris (hydroxymethyl) aminomethane hydrochloride and bovine serum albumin (BSA) were procured from Sigma Chemical Co. (St. Louis, MO, USA).

### 4.2. Preparation of Lizardfish Scales

Fresh lizardfish were obtained from a local fish market in Kota Kinabalu, Sabah, Malaysia. Samples were kept in an insulated box with ice at a ratio of 1:2 (*w*/*w*) to maintain fish freshness. The samples were then transferred to the laboratory of Food Analysis, Universiti Malaysia Sabah. Upon arrival, lizardfish samples were confirmed taxonomically and then prepared for descaling. Descaling was conducted manually with a stainless-steel knife (Brisscoes, Malaysia). The fish scale samples were then washed with tap water. After washing, the samples were put into the polyethylene containers and stored at −20 °C until further experiment.

### 4.3. Preparation of Acid-Extracted Collagens

**Figure 8:**
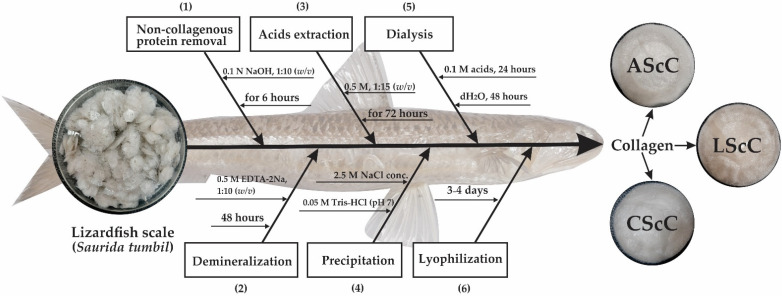
Extraction process of lizardfish scale collagen. Number (1)–(6) described a sequence of extraction steps in producing collagen from lizardfish scale.

Collagen from the scales was extracted with different acids (acetic, lactic, and citric acids). The extraction procedure was as described by Matmaroh et al. [28] with some modifications. For the pre-treatment process, the prepared fish scales were dissolved in 0.1 M NaOH at a ratio of 1:10 (*w*/*v*) for 6 h to remove non-collagenous proteins, and every three hours the alkaline solution was changed. The treated samples were then washed with cold distilled water until completely neutral (pH 7.0). For the demineralization process, the samples were suspended in 0.5 M EDTA-2Na at a ratio of 1:10 (*w*/*v*) for 48 h, and the solution was replaced every 16 h. The suspended samples were rinsed with cold water for 30 min and were periodically changed every 10 min. For the extraction process, the demineralized samples were dissolved in 0.5 M acetic, lactic, and citric acids for 72 h with continuous stirring. Then, the mixtures were filtered through a double-layer of cheesecloth. The supernatants were salted out by adding 2.5 M NaCl plus 0.05 M Tris(hydroxymethyl) aminomethane (pH 7) to stabilize pH during precipitation. The precipitated samples were centrifuged at 15,000× *g* for 30 min at 4 °C. Later, the pellets were dissolved in 0.5 M acids at a ratio of 1:5 (*w*/*v*). The solubilized samples were directly dialyzed using a dialysis tubing cellulose membrane (flat width 43 mm, Sigma) in 20 volumes of 0.1 M acids, followed by cold distilled water for 72 h. After dialysis, the liquid collagens were lyophilized using a freeze-dryer (Labconco, Kansas City, MO, USA). The lyophilized collagens for acetic-, lactic-, and citric-aided extraction were labeled as AScC, LScC, and CScC, respectively, and these collagens were kept in a freezer (−20 °C) until further analyses. All procedures were performed in a cold room (4 °C) and the extraction process is illustrated in Figure 8.

### 4.4. Determination of Yield and Hydoxyproline

Yields of AScC, LScC, and CScC were determined based on the wet weight of scales:

(1) $$\;Yield\;\left(g/100g\right)=\frac{\mathrm{Weight}\;\mathrm{of}\;\mathrm{freeze}-\mathrm{dried}\;\mathrm{collagen}}{\mathrm{Weight}\;\mathrm{of}\;\mathrm{initial}\;\mathrm{wet}\;\mathrm{lizardfish}\;\mathrm{scale}}\;\times \;100$$

For hydroxyproline (Hyp), the method from Bergman and Loxley [61] was used to measure the Hyp content of acid-extracted collagens. First, lyophilized collagens were hydrolyzed in 6 M HCl at 110 °C for 24 h. The hydrolysates were filtered through filter paper (Whatman No. 4). The filtrates were then neutralized with 2.5 M NaOH to reach slightly acidic conditions (about pH 6.0–6.5). After reaching the desirable pH, a total of 0.2 mL of the neutralized samples were transferred into glass test tubes and 0.4 mL isopropanol was added. The mixtures were incorporated with 0.2 mL of oxidant solution and allowed to stand for 3–5 min at room temperature. Next, approximately 2.3 mL of Ehrlich’s reagent solution was added and mixed well. The tubes were subsequently heated at 60 °C for 25 min in a water bath (Memmert, Schwabach, Germany). The heated solutions were then cooled for 5 min in chilled water and diluted to 10 mL with isopropanol. Absorbance against water was measured at 558 nm. The Hyp standard solution (10 to 70 ppm) was also determined.

### 4.5. Analysis of Color Attributes

Analysis of color for AScC, LScC, and CScC was carried out according to the method used by Huda et al. [62], and the collagen from calfskin (CSkC) was used as the standard. The instrument used in this experiment was colorimeter model ColorFlex CX2379 (HunterLab, Los Angeles, CA, USA). The color attributes determined were lightness/brightness (L*), redness (a*), and yellowness (b*). Additionally, the whiteness index (*WI*) for all samples was measured using the following formula [63]:

(2) $$\mathrm{WI}\;=\;100\;-\;{\left[{\left(100\;-\;L^{*}\right)}^{2}\;+\;\left(a^{*2}\right)\;+\;\left(b^{*2}\right)\right]}^{0.5}$$

### 4.6. Sodium Dodecyl Sulfate-Polyacrylamide Gel Electrophoresis (SDS-PAGE)

SDS-PAGE of all acid-extracted collagens from the scales of lizardfish was conducted using the method established by Laemmli [64], and a Mini-PROTEAN electrophoresis system (Bio-Rad Laboratories, Hercules, CA, USA) was used. Approximately 2.5 mg of lyophilized collagens were dissolved in 1 mL of SDS solution (5%) and thoroughly mixed. The mixtures were then heated at 85 °C for 1 h in a water bath (Memmert, Schwabach, Germany). After heating, the collagen samples were subjected to centrifugation at 8500× *g* for 5 min at 24 °C to remove undesirable pellets. The solubilized collagens (15 µL) were transferred into centrifuge tubes with the same volume of sample buffer (0.5 M Tris–HCl, pH 6.8, containing 4% SDS and 20% glycerol) in the presence and absence of 10% β-mercaptoethanol (β-ME). The mixtures were then heated at 85 °C for 5 min. The prepared samples (10–15 µL) were loaded onto a polyacrylamide gel consisting of a 7.5% running gel and 4% stacking gel. The electrophoresis process was set at a constant voltage of 120 V for 1.5 h, and the gel was then fixated for 10 min in the fixation solution, containing 50% (*v*/*v*) methanol and 10% acetic acid. After fixation, the gel was stained for 10 min with 0.05% (*w*/*v*) Coomassie blue R-250 in 5% (*v*/*v*) acetic acid and 15% (*v*/*v*) methanol. The stained gel was further destained with 30% (*v*/*v*) methanol and 10% (*v*/*v*) acetic acid solution. The protein marker (10–250 kDa) was also determined using prestained natural protein standards (dual color standards) (Bio-Rad Laboratories, Hercules, CA, USA).

### 4.7. Ultraviolet-Visible (UV-Vis) Absorption Spectra

AScC, LScC, CScC, and CSkC absorption spectra were scanned using a UV-Vis spectrophotometer (Cary 60, Agilent Technologies, Santa Clara, CA, USA). About 10 mg of freeze-dried collagens were dissolved with 0.5 M acetic acid (1 mL), and the mixtures were transferred into a quartz cell with a light path length of 10 mm. Spectra were determined at wavelengths between 400 nm and 200 nm, with a baseline used in this study of 0.5 M acetic acid solution [14].

### 4.8. Attenuated Total Reflectance–Fourier Transform Infrared Spectroscopy (ATR–FTIR)

ATR-FTIR of the extracted collagens was carried out using an FTIR spectrometer (Agilent Cary 630 FTIR, Agilent Technologies, Santa Clara, CA, USA). Five milligrams of lyophilized collagens were set onto the crystal cell of the spectrometer. The spectra of all extracted collagens were scanned wavenumbers ranging from 4000 cm^−1^ to 1000 cm^−1^ with a spectral resolution of 2 cm^−1^ for 32 scans against a background spectrum recorded from the clean empty cells at room temperature. Spectra data were analyzed using a software program produced by Agilent Microlab. All procedures applied in the present experiment were adopted from Matmaroh et al. [28] with slight modifications.

### 4.9. Analysis of X-ray Diffraction (XRD)

XRD spectra of the lizardfish scale collagen were evaluated with calfskin collagen used as a comparison. XRD analysis was performed according to the procedure by Chen et al. [45]. The lyophilized collagen samples were scanned using an XRD instrument (Rigaku Smart Lab^®^, Akishima, Japan) with copper Kα as the source of X-rays. Voltage and current were fixed at 40 kV and 40 mA, respectively. The scanning range was set to be between 10° and 50° (2*θ*) with a speed of 0.06° per second.

### 4.10. Differential Scanning Calorimetry (DSC)

Thermal stability of AScC, LScC, CScC, and CSkC was evaluated using DSC as described by Kittiphattanabawon et al. [30]. The collagen samples were rehydrated with deionized water at a ratio of 1:40. The rehydrates were then allowed to stand for 48 h in a refrigerator (4 °C). Afterward, the samples were weighed accurately (5–10 mg) in aluminium pans and sealed tightly. Before running samples, the DSC instrument (Perkin-Elmer, Model DSC7, Norwalk, CA, USA) was calibrated using indium as standard. Next, the sealed samples were scanned between a range of 20 °C and 50 °C, with a heating rate of 1 °C/min. An empty pan was prepared for the reference. The maximum transition temperature (*T_max_*) was recorded from the endothermic peak of the thermogram. In addition, Δ*H*, total denaturation enthalpy, was noted by determining the area of the thermogram.

### 4.11. Field Emission Scanning Electron Microscopy (FESEM)

Microstructural properties of extracted lizardfish scale collagens were analyzed by FESEM using a JSM-7900F (JEOL, Tokyo, Japan). Prior to analysis, all freeze-dried samples were sputter-coated for 5 min with gold using a JEOL JFC-1200 (Tokyo Rikakikai Co., Ltd., Tokyo, Japan) fine coater.

### 4.12. Solubility Test at Different NaCl Concentrations and pH Conditions

Solubility of all samples (AScC, LScC, and CScC) at different NaCl concentrations and pH conditions was determined as described by Thuy et al. [15]. For solubility in NaCl, the concentrations selected were between 0 g/L and 60 g/L. Approximately 5 mL of solubilized collagen was transferred into 5 mL of NaCl solution at different concentrations. The mixtures were continuously stirred for 1 h at 4 °C using a FAVORIT Magnetic Stirrer ST0707V2 (Selangor, Malaysia). Then, the stirred samples were subjected to centrifugation at 8500× *g* for 30 min at 4 °C. In terms of solubility at different pH, the prepared acid-extracted collagens were dissolved in 0.5 M acetic acid solution with continuous stirring at 4 °C overnight. Afterward, the mixtures were adjusted to various pH levels (1.0–11.0) using 2.5 N NaOH and 2.5 N HCl solutions. The pH-adjusted samples were allowed to stand for 2 h and centrifuged at 8500× *g* for 30 min in the Eppendorf 5430R Refrigerated Centrifuge (Hampton, VA, USA). Protein content in the solubilized samples was determined using the established method [65] with bovine serum albumin (BSA) as standard. Relative solubility (%) of both treatments was measured using the following equation:

(3) $$\mathrm{Relative}\;\mathrm{solubility}\left(\%\right)=\frac{\mathrm{Current}\;\mathrm{concentration}\;\mathrm{of}\;\mathrm{protein}\;\mathrm{at}\;\mathrm{current}\;\mathrm{NaCl}}{\mathrm{The}\;\mathrm{highest}\;\mathrm{concentration}\;\mathrm{of}\;\mathrm{protein}}\;\times \;100$$

(4) $$\mathrm{Relative}\;\mathrm{solubility}\left(\%\right)=\frac{\mathrm{Current}\;\mathrm{concentration}\;\mathrm{of}\;\mathrm{protein}\;\mathrm{at}\;\mathrm{current}\;\mathrm{pH}}{\mathrm{The}\;\mathrm{highest}\;\mathrm{concentration}\;\mathrm{of}\;\mathrm{protein}}\;\times \;100$$

### 4.13. Statistical Analysis

A completely randomized design (CRD) was used in this study, and experiments were carried out in triplicate. Data were expressed as means ± standard deviation (SD) and a probability value of <0.05 was considered significant. One-way ANOVA was used, and mean comparisons were determined by Duncan’s multiple range tests using SPSS Statistics version 28.0 (IBM Corp., Armonk, NY, USA).

## Figures and Tables

**Table 1:** Yield, Hyp, and total collagen of the lizardfish scale collagen extracted with various acids.

Sample	Yield (g/100 g)	Hyp (mg/g)	Total Collagen (mg/g)	References
**AScC**	0.18 ± 0.03 ^a^	83.29 ± 0.42 ^a^	641.34 ± 0.34 ^a^	This study
**LScC**	0.16 ± 0.04 ^a^	78.39 ± 0.1 ^c^	603.64 ± 0.87 ^c^	This study
**CScC**	0.13 ± 0.02 ^a^	80.94 ± 0.20 ^b^	623.22 ± 1.51 ^b^	This study
**CSkC**	-	91.10	701.47	[18]
**BTcC**	0.05 ± 0.01	87.31	672.29	[8]
**SBcC**	0.38	85	654.50	[27]
**SGcC**	0.46	72	554.40	[28]
**MCcC**	0.64	85	654.50	[18]
**TScC**	0.77	86	662.20	[29]

Values are provided as mean ± standard deviation from triplicate (*n* = 3). Means in the same column with different superscripts are significantly different (*p* < 0.05). AScC: acetic-extracted scale collagen; LScC: lactic-extracted scale collagen; CScC: citric-extracted scale collagen; CSkC: type I collagen from calfskin; BTcC: Bigeye tuna scale collagen; SBcC: Seabass scale collagen; SGcC: Spotted golden goatfish scale collagen; MCcC: Miiuy croaker scale collagen; TScC: Tilapia scale collagen.

**Table 2:** Color parameter of the lizardfish scale collagens extracted with various acids.

Sample	Color Attributes				References
	L*	a*	b*	WI	
**AScC**	79.94 ± 0.06 ^a^	1.41 ± 0.15 ^c^	3.67 ± 0.12 ^a^	79.56 ± 0.58 ^a^	This study
**LScC**	76.25 ± 0.11 ^b^	3.11 ± 0.40 ^a^	8.21 ± 0.58 ^a^	74.68 ± 0.12 ^c^	This study
**CScC**	79.52 ± 0.36 ^a^	2.15 ± 0.15 ^b^	4.80 ± 0.57 ^a^	78.86 ± 0.36 ^b^	This study
**CSkC**	78.93 ± 0.59 ^a^	−0.07 ± 0.03 ^d^	1.42 ± 0.27 ^b^	78.88 ± 0.58 ^b^	This study
**BSkC**	65.41 ± 0.08	0.14 ± 0.01	3.16 ± 0.03	65.27	[35]
**SSkC**	89.49 ± 0.28	−0.30 ± 0.01	5.60 ± 0.13	88.09	[36]

Values are provided as mean ± standard deviation from triplicate (*n* = 3). Means in the same column with different superscripts are significantly different (*p* < 0.05). AScC: acetic-extracted scale collagen; LScC: lactic-extracted scale collagen; CScC: citric-extracted scale collagen; CSkC: type I collagen from calfskin; BSkC: barramundi skin collagen; SSkC: snakehead fish skin collagen.

**Table 3:** Fourier transform infrared spectroscopy peak area and the assignment for collagens from the scale of lizardfish.

Peak Area				Peak Assignment	References
AScC	LScC	CScC	CSkC		
3276.41	3296.91	3285.73	3309.01	Mainly N-H stretching coupled with hydrogen bond (Amide A)	[46]
2931.62	2935.35	2926.03	2921.47	CH_2_ asymmetric stretching (Amide B)	[47]
1628.89	1628.89	1628.89	1635.87	C=O stretching/hydrogen bond coupled with COO- (Amide I)	[48]
1541.29	1541.29	1541.29	1542.71	N-H bend coupled with C-N stretching (Amide II)	[48]
1235.64	1235.64	1237.51	1237.15	N-H bend coupled with C-H stretching (Amide III)	[48]

AScC: acetic acid-extracted collagen; LScC: lactic acid-extracted collagen; CScC: citric acid-extracted collagen; CSkC: Commercial calf skin collagen.

## Data Availability

The data presented in this study are available upon request from the corresponding author.
